# Review of the Management of Relapsed/Refractory Follicular Lymphoma: An Italian Perspective

**DOI:** 10.1002/hon.70140

**Published:** 2025-10-07

**Authors:** Giacomo Loseto, Maria Chiara Tisi, Antonella Anastasia, Alessandro Broccoli, Ilaria Del Giudice, Caterina Patti, Benedetta Puccini, Caterina Stelitano, Vittorio Zilioli, Stefano Luminari

**Affiliations:** ^1^ Hematology and Cell Therapy Unit IRCCS Istituto Tumori “Giovanni Paolo II” Bari Italy; ^2^ Hematology Unit San Bortolo Hospital Vicenza Italy; ^3^ Department of Hematology ASST Spedali Civili Brescia Italy; ^4^ IRCCS Azienda Ospedaliero‐Universitaria di Bologna Istituto di Ematologia “Seràgnoli” Bologna Italy; ^5^ Dipartimento di Scienze Mediche e Chirurgiche Università di Bologna Bologna Italy; ^6^ Hematology Department of Translational and Precision Medicine Sapienza University Rome Italy; ^7^ Oncohematology Unit A.O.O.R. Villa Sofia Cervello Palermo Italy; ^8^ Hematology Unit Careggi University Hospital Florence Italy; ^9^ Hematology Grande Ospedale Metropolitano Bianchi‐Melacrino‐Morelli Reggio Calabria Italy; ^10^ Division of Hematology ASST Grande Ospedale Metropolitano Niguarda Milan Italy; ^11^ Division of Hematology Azienda Unità Sanitaria Locale‐IRCCS Reggio Emilia Reggio Emilia Italy; ^12^ Chimomo Department University of Modena and Reggio Emilia Reggio Emilia Reggio Emilia Italy

**Keywords:** bispecific antibodies, follicular lymphoma, immunotherapy CAR T‐cells, management, relapsed/refractory

## Abstract

Follicular lymphoma (FL) is an indolent disease characterized by multiple relapses and eventual refractoriness to therapy. Despite advancements in therapeutic approaches, FL treatment algorithm and management remain not well‐established, necessitating both ongoing research into novel therapeutic strategies and in stating patient journey. We propose a comprehensive overview of current standard treatments for relapsed or refractory (R/R) FL, including chemoimmunotherapy and stem cell transplantation, and insights into emerging therapies such as chimeric antigen receptor (CAR) T‐cell therapy and bispecific antibodies. We discuss the efficacy and safety profiles of these innovative treatments, their integration into the therapy armamentarium, and the potential they hold in altering the natural history of FL. Additionally, we propose a therapeutic flow depending on POD24 (i.e., progression of disease within 24 months), transformed disease, early relapse and fast/low progression, with the aim to provide a useful tool to all physicians dealing with this disease for achieving sustained remission and improving the quality of life in patients with R/R FL.

## 1 Introduction

Follicular lymphoma (FL) is the second most frequent subtype among non‐Hodgkin lymphomas (NHL), accounting for 20%–30% of cases [1]. Although characterized by an indolent clinical course and excellent response to treatment, FL is still considered incurable, and patients are at continuous risk of disease recurrence. Overall, most patients with FL show very long survival which is now measured in decades and in some cases a FL diagnosis has very limited impact on life expectancy allowing to achieve a status of functional cure. Current unmet clinical needs for FL are mainly represented by the uncertainties regarding the ability to accurately predict patients' outcomes for any line of therapy, by the risk of transformation into an aggressive lymphoma, and by the risk of early and by multiple relapses [2, 3, 4, 5, 6, 7, 8, 9, 10]. Moreover, considering the excellent outcomes of FL patients, a relevant unmet need is represented by the prevention of long‐term complications, mainly represented by infections and second malignancies, and for which a fine tuning of the treatment choice at each line of therapy should be identified as one of the main therapeutic issues [11]. In this scenario the availability of novel therapeutic options with an optimized safety and efficacy profile are eagerly warranted.

Regarding our ability to predict patients' outcomes at the time of initial diagnosis several prognostic factors have been validated in the last decades. Among them FLIPI, FLIPI2 and PRIMA‐PI are the most frequently used [12, 13, 14]. Novel prognostic factors have been validated in FL, which incorporate molecular features functional imaging parameters such as Total Metabolic Tumor Volume, or metabolic response assessed by positron emission tomography (PET) with 2‐[(18)F]fluoro‐2‐deoxy‐D‐glucose [FDG], FDG‐PET, but none of them were accurate and stable enough under different therapeutic modalities to support clinicians [15, 16, 17, 18, 19].

For many years the use of chemotherapy has represented the main backbone of FL treatment and today is recommended, in combination with anti‐CD20 monoclonal antibodies, for patients with high tumor burden and/or symptomatic subjects [19]. With the advent of immunochemotherapy in first line an excellent control of the disease was observed, with approximately 90% of patients achieving a complete response (CR). Once first therapeutic approach is completed, anti‐CD20 maintenance therapy can be prescribed with the aim of prolonging duration of remission and to reduce the risk of relapse within 24 months (POD24) [20]. Even if imaging (FDG‐PET) or molecular techniques measuring minimal residual disease are available to identify patients with the best quality of response, these parameters cannot be recommended to adapt post induction therapy as shown by the FOLL12 study [21]. Overall, with the appropriate first‐line therapy, patients can achieve a median progression‐free survival (PFS) longer than 8 years [22].

Management of a patient responding to first‐line therapy should be tailored to identify events which may anticipate highest risk features. We support the ESMO guidelines recommendations which mandate strict clinical monitoring suggesting an optional radiological follow‐up every 6 months for the first 2 years after the end of first line, then every 12 months until the fifth year [19]. In this scenario FDG‐PET does not play a role as mandatory follow‐up procedure, while it is highly recommended in patients with suspected/identified disease recurrence to confirm relapse or to rise a suspicion of transformation (tFL) identifying target sites for biopsy, which should be always considered, when feasible.

Regarding relapsed refractory (R/R) patients, once tFL has been excluded, few prognostic factors were singled out and no prognostic model has been yet validated to support clinical and therapeutic decisions. POD24 is associated with a high risk of death,^7^ however there is a general consensus that management of R/R patients cannot be only based on one single parameter like POD24, even if strongly prognostic [7, 19]. Applying an approach based on disease stage and tumor burden like the one used in newly diagnosed patients seems to this Expert Panel a good suggestion. This approach would better account for the heterogeneous outcomes of R/R FL allowing physicians to choose among several alternative options, observation for asymptomatic cases, considering local management for localized relapses, and systemic therapy in case of high tumor burden [23, 24, 25]. Even if strongly associated with the risk of death, also POD24 patients show heterogeneous outcomes, and when an effective salvage therapy is used, POD24 patients may reduce their initially foreseen bad outcomes [26]. A more modern definition of a high‐risk FL should be then applied to patients who are subjected to subsequent relapses, each with short duration of remissions and/or to patients who experience transformation and/or short duration of response (DoR) to the last therapy.

### 1.1 Second Line of Therapy

Patients with R/R FL will experience progressively shorter DoR to subsequent treatments [27]. Despite improvements in overall survival (OS) following anti‐CD20 antibody‐containing therapies, lymphoma remains the leading cause of death for FL patients [11].

Following ESMO recommendations for second‐line treatment it is remarkable to remember that, when a relapse or progression occurs, the first challenge to consider is the possibility of histological transformation (about 5%/year in the rituximab era) [5, 19]. This dramatic event should be early detected, especially in the presence of B symptoms, rapid deterioration of clinical conditions, or areas of high standardized uptake values at FDG‐PET. As already stated, this latter parameter should be used to direct the optimal target site of biopsy for histological confirmation, when feasible.

When treatment is required, there is a lack of consensus on second‐line approach for R/R FL patients, and no chemotherapeutic program among those available has proved superior over others [28]. The several available treatment choice options may range from radiotherapy (in early‐stage cases), rituximab alone (in low tumor burden), chemotherapy combination or oral immunomodulating drug plus rituximab, and, for selected patients, autologous stem cell transplantation (ASCT).

The assessment of prior therapy is important to avoid the use of cross‐resistant regimen with chemo‐immunotherapy or chemo‐free regimen (*R* ^2^). In primary rituximab CHOP‐refractory cases or remissions lasting less than 6 months, rituximab could be avoided and replaced with obinutuzumab as suggested by the Gadolin study which proved better efficacy of the obinutuzumab and bendamustine combination versus bendamustine alone [29]. However, in the era of T‐cell engaging therapies in FL, available from third line and beyond, the use of bendamustine as second‐line treatment should be cautiously considered to avoid damage of the T‐cell compartment [30].

There is a consensus that, for patients who experience an early progression of disease, ASCT should be used in eligible patients thanks to better results on outcome compared to standard chemo‐immunotherapy, especially if performed within 1 year from first treatment failure and in first or second CR [31, 32, 33]. However, the use of ASCT in R/R patients is supported by a wide number of retrospective studies, mostly published in the pre‐rituximab and pre‐FDG‐PET era, with discordant results, and ASCT has rarely been compared directly to novel treatments [34, 35, 36]. Ignoring all the variabilities of the studies, it can be observed that 5‐year PFS after ASCT is around 50%–60%, and 5‐year OS is reported with a wide range from 56% to 94% [37, 38]. Although most of these results cannot be simply transferred to the current panorama, one important finding is the potential of ASCT to induce long‐term remissions in a subgroup of patients [31, 32]. In a large retrospective analysis reported from the GELTAMO group, the apparent plateau in PFS curves after 16 years for patients who underwent transplantation in CR status, with 59% of patients in continuous CR suggests that a substantial number of these patients may never relapse [39]. The most relevant prognostic variable in this series was disease status at high‐dose therapy (HDT)ASCT, a well‐established factor [40, 41, 42, 43, 44]. Nevertheless, the occurrence of second malignancies is a concern following HDT/ASCT, with reported incidences varying widely among retrospective and prospective studies and is an argument against this therapeutic approach for some Hematologists [45, 46, 47, 48, 49].

Recently the multicenter, randomized, phase III FLAZ12 trial compared anti‐CD20 radioimmunotherapy (RIT) with ASCT as consolidation after chemo‐immunotherapy, both followed by rituximab maintenance [50]. This study did not demonstrate the superiority of ASCT versus RIT but in the ASCT cohort more toxicities occurred. According to subgroup analysis, only in a small population with refractory disease, ASCT was associated to a better outcome than RIT (HR 0.11 vs. 1.27, interaction *p* = 0.001). Besides, the overall results showed a continuous tendency to relapse after ASCT (3‐ and 6‐year PFS rate of 60% and 44%, respectively) [50].

To date, defining the role of HDT/ASCT in FL remains a challenge, partly because most available treatment options are associated with excellent outcome, and partly because many clinical studies have short follow‐up or lack of statistical power to state OS robust results.

With the advances in new drugs against FL, these present recommendations could have a limited period of validity. There is a general agreement that ASCT could be considered as an option for the second‐line therapy only in young patients with high‐risk features and short DoR to first‐line therapy. In these cases however consolidation with ASCT after reinduction therapy should be indicated only upon previous confirmation of metabolic CR.

Several novel therapeutic options beyond the commonly used immuno‐chemotherapy regimens have been explored over the last decades but few of them are currently available/approved. Among novel agents the most studied and effective drug is lenalidomide, the prototypical oral immunomodulating drug [51].

Lenalidomide, was initially studied in phase II study either as single agent or in combination with rituximab (the *R* ^2^ regimen). A first randomized study showed better response rates and prolonged PFS with the *R* ^2^ combination versus single agent rituximab [52]. The larger phase III AUGMENT trial compared *R* ^2^ versus rituximab plus placebo in R/R FL and marginal zone lymphoma (MZL) [53]. A total of 358 patients were randomized (*n* = 178 *R* ^2^ arm; *n* = 180 control arm); at a median follow‐up of 65.9 months, median PFS 27.6 months for *R* ^2^ cohort versus 14.3 months for control arm, with HR = 0.50 (95% CI, 0.38–0.66; *p* < 0.0001). Although median OS was not reached for either group, there was an improvement in OS for the *R* ^2^ arm (HR = 0.59, 95% CI, 0.37–0.95, *p* = 0.0285) [54]. Based on these results, the FDA approved *R* ^2^ for R/R FL.

Lenalidomide has also been combined with other agents confirming itself as a good candidate for combination chemo‐free regimens. In the phase II Galen study lenalidomide was combined with Obinutuzumab [55]. After a median follow‐up of 2.6 years, the overall response rate (ORR) was 95% with 2‐year PFS of 65% and OS of 87%, respectively.

More recently, lenalidomide has been combined with both anti‐CD20 and anti‐CD19 monoclonal antibodies in the inMIND study. The trial compared the three drug combinations with *R* ^2^ in 546 R/R FL most of whom received only one prior line of therapy. The experimental combination was associated with a 60% reduction of the risk of progression without a significant increase in toxicity. While study follow‐up is still immature at the time of first data disclosure, a trend for OS improvement was observed [56]. Lenalidomide was also combined with mosunetuzumab and compared with standard salvage therapies in the Celestimo randomized trial, after promising results in a small phase II study [57]. Finally, in the ongoing Zuma22 trial which compared the use of axicabtagene cloleucel (axi‐cel) with standard therapy in R/R FL, a cohort of second‐line patients with POD24 was also included allowing to assess the role of ASCT in this setting.

In conclusion, looking forward to the disclosure of results from ongoing trials and to the registration of novel agents (alone or in combination), current available data strongly support the use of the *R* ^2^ as preferred option for the second‐line therapy of R/R FL. The preferred sequencing of therapy in R/R FL yet remains to be defined and shared.

### 1.2 Third Line of Therapy and Beyond

Over the past decade, phosphatidylinositol 3‐kinase (PI3K) inhibitors and *R* ^2^ were approved [53, 58], for the treatment of multiple relapsed patients but efficacy of these treatments is reduced when administered in later lines [10]; moreover, treatment interruptions and discontinuations of PI3K due to toxicities have been frequently observed and prompted to a global retraction of this class of agents in previously approved indications [59].

In the third‐line setting and beyond, autologous anti‐CD19 CAR T‐cells cell therapy have been recently approved. The efficacy and safety of tisagenlecleucel (tisa‐cel) after two or more lines in adult R/R FL patients was explored in the phase II trial ELARA [60]. This study included 97 patients, with a median number of prior lines of therapy of 4 (range, 2–13); more than 50% of patients had a high‐risk feature and 64.9% had POD24. 45 (46%) patients received bridging therapy (BT). The rate of responses in terms of CR was 69.1% and the ORR was 86.2%. Within 8 weeks of infusion, rates of cytokine release syndrome (CRS) were 48.5% (grade ≥ 3, 0%) and immune effector cell‐associated neuro‐toxicity syndrome (ICANS) 4.1% (grade ≥ 3, 1%), with no treatment‐related deaths. In a recent update, after median follow‐up of 53 months, median PFS was 53.3 months and median OS was not reached [61]. Patients in CR demonstrated better efficacy than the one of the ELARA whole study population for each of these endpoints. Of note, by month 6, the probability of resolution of any cytopenia was 82.0% [62]. Efficacy and durable responses were seen also in patients with high‐risk baseline disease characteristics. In particular for patients with POD24, ORR was 82% and CR rate 59%; 48‐months PFS and OS were 45.5% and 80.8%, respectively; 48‐months PFS was 45.5% (high FLIPI), 45.2% (bulky disease), 52.8% (double refractory), showing a comparable long‐term efficacy also in these high‐risk subgroups. Tisa‐cel results continued to demonstrate highly durable efficacy and a favorable safety profile [61].

The ZUMA‐5 single‐arm, multicenter, phase II trial evaluated the role of axi‐cel in indolent lymphomas including FL [63]. One hundred twenty‐four patients with FL received axi‐cel infusion: the median number of prior lines of therapy was 3 (2–4), 55% had POD24; 68% were refractory to the last prior antineoplastic treatment. BT was administered to four patients. Among the 84 patients with FL eligible for the primary analysis, 94% had an ORR, with 79% having a CR. CRS occurred in 78% patients with FL (grade ≥ 3,6%), neurological events occurred in 56%, (grade ≥ 3, 15%). With the updated median follow‐up of 65.7 months,^64^ median PFS was 57.3 months, and median OS was not reached. Sixty‐months PFS rates were consistent regardless of high‐risk characteristics, including POD24.^64^ Patients with FL who received prior bendamustine had a lower 36‐months PFS rate compared to those who did not receive bendamustine, in particular if bendamustine was administered at maximum 6 months before leukapheresis [64]. Grade 3 or worse cytopenias occurred on day 30 in 33% of patients and in few patients was persistent at 12 and at 24 months after infusion [64].

The phase II TRANSCEND FL study evaluated lisocabtagene maraleucel (liso‐cel) in patients with R/R FL, including second‐line patients who all had POD24 from diagnosis [65]. Among population in third line and beyond (103 patients), 57% had high‐risk FLIPI, 43% of patients had POD24, 53% were symptomatic and 64% were double refractory [66]. In third line or later FL (*n* = 101), ORR was 97% and CR rate was 94%. With the recent update (median follow‐up 30 months) the median DoR, PFS and OS were not reached: 24‐months PFS and OS were 72.5% and 88.2%, respectively. ORR, CR rate, DoR and PFS remained high across patient subgroups, including the high‐risk ones, including POD24 [67]. Any‐grade CRS occurred in 59%, with grade 3 in only one patient and no grade 4 or 5 events. Neurological events occurred in 11% of patients (grade 3, 2%), and no grade 4 or 5 events occurred. Prolonged cytopenia (grade ≥ 3 cytopenias based on laboratory values at day 29) was reported in 22% of patients.

These interesting and promising data on the role of CAR T‐cells in FL from trials were confirmed also in real‐world experiences encouraging about the use of CAR T‐cell treatment in R/R FL [63, 68].

Neverthless, the optimization of BT remains undefined and, given that FL is an indolent and biologically complex disease, with a typical long course, probably still longer follow‐up is needed to confirm the long‐term efficacy and to define the cure for responding patients with this approach.

For the third line or later, mosunetuzumab, the first‐in‐class CD20xCD3 T‐cell engaging bispecific antibody (BsAb), received the regulatory approval in our Country in September 2023.

This is an off‐the‐shelf medication consisting of a fixed‐duration administration, with step up dosing during the first cycle. In the pivotal phase II study, mosunetuzumab demonstrated an ORR of 80%, with a CR rate of 60% in a cohort of 90 heavily pretreated patients affected by R/R FL who received a median of 3 previous lines (range, 2–10) [69]. Among these patients, 68.9% were refractory to their last prior therapy and 52.2% of the patients displayed POD24. According to the most recent updates with a median follow‐up of 49.4 months median PFS was 24 months and median OS was not reached; median DoR and duration of complete response were 46.4 and 51.8 months, respectively. Thirty‐five (64.8%) patients with a CR remained in remission at 4 years [70].

Looking at the POD24 cohort median PFS was 21.7 months and median OS was not reached [70].

Higher efficacy was documented in patients receiving mosunetuzumab earlier in the course of the disease [71].

As far as age is concerned, higher response rates were observed in patients older than 65 years, without survival differences between the age groups [71].

According to the recent update of the phase I trial, an attempt of retreatment in patients with a late relapse to mosunetuzumab is feasible, but cases reported poor sample size (8 patients) to draw robust conclusions [62, 72].

To date, CRS represents the most significant mosunetuzumab‐related adverse event (AE), with the highest incidence on cycle 1, day 15. 44.4% of patients, grade 3–4 1.1% [73].

Neutropenia was observed in 28.9% of the patients (grade 3–4 26.7%), without any febrile neutropenia event and serious infections were reported in 20% of the overall study population, with most of serious infections within the first four cycles [69, 71].

Due to the mechanism of action of BsAbs, lymphopenia and reduction in the concentration of immunoglobulins (Ig) represent a major concern and support with Ig formulations should be considered in patients receiving mosunetuzumab [71]. Of note, the recovery of Igs occurs within 12 months, unlike what was observed in patients treated with CAR T‐cells [62].

Like mosunetuzumab two other anti‐CD20‐CD3 bispecific antibodies have been studied in R/R FL treated in third line or beyond. Odrontextamab was evaluated in a phase II study with 128 patients. This agent led to high rates of deep response with durability in R/R FL, with 73% of patients attaining CR [74]. Epcoritamab was evaluated in a phase II study with 128 patients. Preliminary analysis reported an ORR of 82.0%, with a CR rate of 62.5% [75]. Different from mosunteuzumab schedule both odronextamab and epcoritamab are administered until progression or unacceptable toxicity and required a prolonged step‐up dosing, with the addition of split dosing and with mandatory hospitalization for odronextamab.

In addition to the novel T‐cell engaging agents, a promising, more conventional, new therapy for R/R FL is represented by the combination of zanubrutinib and obinutuzumab (ZO) [76]. The ROSEWOOD study showed an increased PFS with ZO (28.0 months) compared to obinutuzumab alone (10.4 months). The overall ZO safety profile was found to be manageable. The most common severe side effects (grade ≥ 3) were neutropenia, thrombocytopenia, pneumonia, COVID‐19 infection and anemia [76].

In summary several novel options have shown promising efficacy data in the treatment of R/R FL from third line and later. In several countries CAR T‐cells (tisa‐cel) and BsAbs (mosunetuzumab) have both indications and are reimbursed for the third‐line therapy of FL while axi‐cel can only be prescribed from fourth line. Several other agents for combinations (liso‐cel, odronextamab, epcoritamab, ZO) have already been approved by EMA but are in the process of being approved for local reimbursement. In this scenario of highly effective option in overlapping indications and lacking direct comparative studies, it is important to highlight some specific features and characteristics of three agents to help physicians in the correct choice. Several dimensions should be considered while trying to define different patient profiles, including but not limited to efficacy and safety data (Table 1), ease of use, treatment duration, patients preferences, resource allocation, capacity of treatment center, and sustainability.

**TABLE 1:** Clinical trials that led to drugs approval in Italy for relapsed7refractory follicular lymphoma.

	ELARA (*n* = 97)	ZUMA‐5 (*n* = 127)	GO29781 (*n* = 90)
	Tisagenlecleucel [60]	Axicabtagene ciloleucel [63]	Mosunetuzuamb [69]
Characteristic			
Median age (range), years	57 (29–73)	60 (34–79)	60 (29–90)
Stage III–IV disease, *n* (%)	85.6%	86%	76.7%
≥ 3 FLIPI, *n* (%)	59.8%	44%	44%
Bulky disease	64.9%	51%	
Median no. of prior therapies (range)	4 (2–13)	3 (2–4)	3 (2–10)
Prior PI3Ki therapy, *n* (%)	14.4%	28%	18.9%
Refractory disease to last line of therapy, *n* (%)	78.3%	68%	68.9%
Double refractoryd: Anti‐CD20 mAb + alkylating agent (%)	68%	44%	78.9%
POD24, *n* (%)	64.9%	55%	52.2%
Prior ASCT, *n* (%)	36.1%	24%	21.1%
Efficacy			
ORR	86.2%	94%	80%
CR	69.1%	79%	60%
PFS	48‐months PFS 50.2%	Estimated 60‐months PFS: 49.8%	Estimated 4‐years PFS rate: 38.6%
DoR	Estimated 24‐months DOR: 66.4%	Estimated 60‐months DOR: 52.2	Median DOR: 46.4 months; median DOCR: 51.8 months
OS	48‐months OS: 79.3% in efficacy‐evaluable pts	Estimated 60‐months OS: 68.9	Estimated 4‐year OS rate: 82.7%
PFS (with POD24 vs. without POD24)	36‐months PFS: 50% with POD24 versus 59% without POD24;	36‐months PFS: 59% with PDO24 versus 52% without POD24	36‐months PFS:44% with PDO24 versus 42% without POD24. Estimated 4‐years PFS: 38.9%
OS (with POD24 vs. without POD24)	36‐months OS: 83% with PDO24 versus 81% without POD24; 48‐months OS: 80.8% with POD24		36‐months OS: 84% with PDO24 versus 81% without POD24. Estimated 4‐years OS: 86.4%
Safety			
CRS (all/G ≥ 3)	48.5%/0	78%/6%	44%/1,1%
neurological events (all/G ≥ 3)	37.1%/3%	56%/15%	2.2%/0
ICANS (all/G ≥ 3)	4.1%/1%	0	0

Abbreviations: ASCT, autologous stem cell transplantation; CR, complete response; CRS, cytokine release syndrome; DoCR, duration of complete response; DoR, duration of response; G, grade; ICANS, immune effector cell‐associated neuro‐toxicity syndrome; ORR, overall response rate; OS, overall survival; PFS, progression‐free survival; POD24, progression of disease within 24 months.

It should be also considered that in FL, for its chronic course, the issue of the best sequencing remains relevant and still open, until we have strong evidence of a potential cure.

We conceived the following paragraph starting form literature evidence to identify some recommendations/insights agreed among our Expert Panel which may support treatment decisions in different FL patient population. The methodology consisted in both Expert Panel discussions and structured consensus meetings. These recommendations are mainly focused on the choice between BsAbs and CAR‐T in the third line or later setting and have been generated in a country specific context but can be easily transferred to other similar health system organizations.

### 1.3 Expert Panel Recommendations/Insights

1. Patient profile.
  – *CAR T‐cells are preferred for fit, early relapsed, high‐risk FL patients.* Both patient's features (age, fitness) and disease features (transformation, length of remissions, prior therapies) should be considered.
  – BsAbs *offer a more accessible option* for a wider patient population, including those unsuitable for CART.
2. Prior therapies
  – *CAR T efficacy may be influenced by the number and type of prior treatments*, due to impacts on T‐cell quality.
  – BsAbs *demonstrate robust activity regardless of treatment history*, making them a more flexible option in later lines of therapy.
3. Center experience.
  – *CAR T demand high specialization and accredited infrastructure.*
  – BsAbs *are more broadly accessible*, requiring moderate experience with immune‐related adverse events.
4. Treatment duration.
–
*CAR T offers a short, one‐time treatment*, with intensive monitoring afterward.
  – BsAbs *require ongoing dosing over several months.* Among available BsAbs, mosunetuzumab is administered with a fix duration of 6 or 12 months depending on response while others are administered until progression or toxicity.
5. Acute toxicity.
  – *CAR T has more severe and resource‐intensive acute toxicities.*
  – BsAbs *have milder, more manageable profiles.*
6. Patient management.
  – *CAR T require intensive inpatient management.*
  – BsAbs *allow for outpatient administration with manageable monitoring*, making them more accessible and less resource‐intensive.
7. Long term toxicity.
  – *CAR T may carry more persistent immune‐related toxicities.*
  – BsAbs *tend to have fewer and more reversible long‐term effects*, though both require infection monitoring.
8. Patient preferences.
  – Patients seeking an aggressive, potentially one‐time therapy may lean toward *CAR T*, while those prioritizing *convenience, safety, and outpatient care* often prefer BsAbs. Shared decision‐making, considering personal values, clinical status, lifestyle, proximity to the hospital and caregiver availability,
9. Sustainability.
  – *CAR T offers high efficacy but with significant sustainability challenges* due to its individualized, resource‐intensive nature.
  – BsAbs *are more sustainable* in terms of logistics, scalability, and system‐wide implementation, supporting broader and more equitable access over time.

**FIGURE 1:**
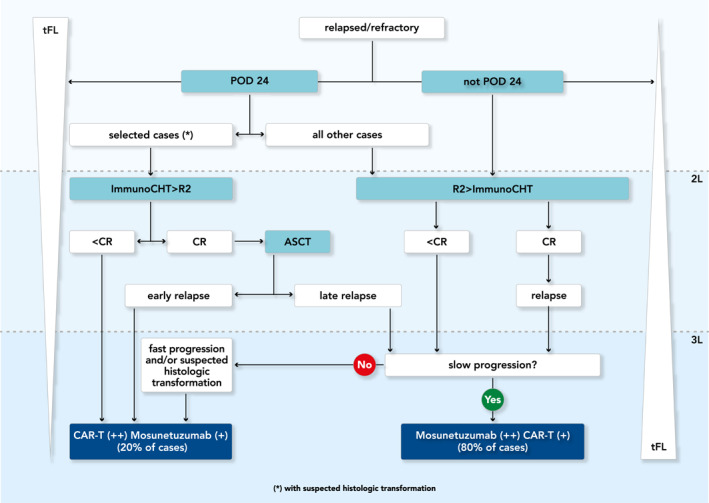
Flow‐chart for the management of relapsed/refractory follicular lymphoma R/R FL patients. “+” represents preferences for therapy if applicable, that is ++ I preferable than +. 2L, second line of therapy; 3L, third line of therapy; ASCT, autologous stem cell transplattaion; CAR T, chimeric antigen receptor T‐cells therapy; CR, complete response; immmunoCHT, immunochemotherapy; POD24, progression of disease within 24 months; R2, lenalidomide plus rituximab; tFL, transformed follicular lymphoma.

Based on the knowledge and opinions reported above, we propose a flow‐chart for R/R FL patients (Figure 1).

## 2 Conclusions

The treatment of R/R FL is experiencing a paradigm shift from conventional cytotoxic therapies to novel chemo‐free options. The transition to chemo‐free therapies has already been implemented starting from second‐line therapies and a plethora of option is currently available from third line and beyond. CAR T (tisa‐cel) and BsAbs (mosunetuzumab) have both indications for third line FL in Italy. Both drug classes are highly active, and their safety profiles partially overlap, even if the lower rate and quality of severe events favors BsAbs.

Notably, patients could receive both classes of therapies in sequence; however, data guiding this decision are still sparse.

Considering the 3 most advanced agents in each class, BsAbs seem to this Expert Panel a better option than CAR T as the third‐line standard of care treatment for the typical patient with R/R FL [56]. Conversely, we suggest prioritizing CAR‐T for patients with proven or suspected histologic transformation given the curative‐potential of this approach based on trial data from R/R diffuse large B‐cell lymphomas and for cases who get to third‐line therapy in a short period oof time from initial diagnosis (Figure 1).

Logistical aspects, availability and treatment Center accessibility, patient comorbidities and preference, and previous treatment, are also key aspects to be considered when choosing treatment sequencing.

Moving immunotherapeutic strategies into earlier lines of treatment is the desirable future prospect for this disease.

## Author Contributions

S.L. and G.L. conceived the Review, finalized the final draft and supervised the Panel work and contributions. All Authors contributed to the draft of the paper also with pro‐active discussion, edited the draft and approved the final version.

## Conflicts of Interest

S.L. declared Consulting from Roche, Kite, BMS, Novartis, Abbvie and Incyte, Speakers’ bureau from Roche, Kite and BeiGene and Travel from Roche and BeiGene; I.D.G. declared Speakers’ bureau and Advisory board from Takeda, Roche, Janssen, AstraZeneca, and BeOne; V.Z. declared Speakers’ bureau and Advisory board from Abbvie, AstraZeneca, BeiGene, Incyte, Janssen, Kite, Lilly, Novartis, Roche, Sobi and Takeda; M.C.T. declared Advisory board from Incyte, BMS, Gilead Science, Novartis, Beigene, Roche, Abbvie and Speakers Bureau from Incyte, Roche, Gilead Science, Novartis, Janssen, Lilly, Sobi, BMS and AstraZeneca; G.L. declared Consultancy, Advisory board and Speaker bureau from Janssen, Roche, Kite/Gilead, Italfarmaco, Takeda, AbbVie, Incyte, Astrazeneca, Beigene, GSK and Lilly. The other authors have no conflicts of interest to disclose.

## Peer Review

The peer review history for this article is available at https://www.webofscience.com/api/gateway/wos/peer-review/10.1002/hon.70140.

## Data Availability

The authors have nothing to report.
